# Umbilical Hernia in the Adult

**Published:** 1879-01

**Authors:** William Warren Potter

**Affiliations:** Batavia, N. Y.


BUFFALO

### Medical and Surgical Journal.

VOL. XVIII. JANUARY, 1879. NO. 6.

### Original Communications.

:o:

*ART. I.—Umbilical Hernia in the Adult.* By WILLIAM WARREN POTTER, M. D., of Batavia, N. Y.

A paper read before the Medical Association of Central N. Y. at Rochester, Nov. 19, 1878.

That species of rupture in which the abdominal viscera are protruded through the round opening of the *linea alba* which transmits the umbilical blood-vessels of the fœtus, has been named umbilical hernia. The terms *Exomphalos* and *Omphalocele* have also been applied to the malady; *exomphalos* especially being quite frequently used by many of the older authors. Mr. .Samuel Cooper* remarks that "custom appears to sanction the extension of the terms *exomphalos,* omphalocele, and umbilical hernia, not only to protrusions of the bowels through the opening of the navel, but to all other tumors of a similar nature which present themselves anywhere in the vicinity of the aperture, and the majority

*First Lines in Surgery. Ed. of 1822. Vol. II, p. 2.

of which actually take place in the *linea alba* either above or below the precise situation of the umbilicus."

Sir William Lawrence * asserts that the term *exomphalos* can be applied with propriety to that rupture only which occurs at the umbilicus, while any displacement of the viscera through the linea alba, in the neighborhood of the navel, should be classed with ventral hernia.

J. L. Petit was the first observer to point out the distinction between true *exomphalos*, and hernia of the *linea alba* in the immediate vicinity of the umbilicus; for before his time, says Mr. S. Cooper,† all umbilical hernia were supposed to protrude directly through the navel. The question appears to have been a point of controversy among writers at the close of the last, and beginning of the present century, some holding to the one view and others of equal acumen assisting the contrary opinion. The opinion of Sir Astley Cooper is quoted in support of the view, that umbilical hernia usually takes its course through the navel aperatnre. Sir William Lawrence ‡ seems to have reached the gist of the matter when he declares "that the anatomical facts will furnish us with two pathological inferences. First; that infancy is more subject than any other age to umbilical hernia, properly so called, when the viscera are protruded through the navel itself. Secondly; that adults may be more exposed to that species of the complaint in which the hernia takes place in the vicinity of the umbilicus." And again, the same author asserts § that whether the protrusion takes place most frequently in the former or in the latter of these two situations (that is, directly through the navel or in the immediate vicinity of that part), can be a matter of no *practical* consequence whatever, although it might perhaps influence the name of the complaint.

To acquire an accurate knowledge of the malady now under consideration, it should be studied under its three principal forms: first, congenital *exomphalos;* second, *exomphalos* of the young subject; and finally, umbilical hernia in the adult. It is with the latter sub-division of the disease that we have to do, more espe-

* Lawrence on Ruptures, p. 407.

† First Lines. Vol. II. p. 9.

‡ Lawrence on Ruptures, p. 108.

§ Ibid, p. 407.

cially, to-day; and, with your permission, I will here introduce a brief clinical history of a case of strangulated umbilical hernia, which, not very long ago, happened to fall under my observation. After supplimenting the account with some remarks pertaining especially to the case itself, I will submit some observations germain to the whole subject.

CASE.—Mrs. C. residing near Corfu, Genesse county, aet 39 years, height 4 feet 10 inches, weight 195 pounds, very obese, married, mother of two children aged ten and eight years respectively, was, on the 19th of January 1877, at seven o'clock A. M., seized with great distress in the umbilical region, which she readily referred to the descent of an old hernia. The tumor had often before given her similar trouble, and as often had she been able to reduce it herself; but now, finding her efforts in that direction unavailing, she summoned her physician, a homeopathic practitioner residing a mile away. He soon reached her bedside and remained in constant attendance until five o'clock P. M. without, however, affording his patient any substantial relief. Her sufferings had now become so extreme as to alarm the friends, and Dr. A. H. Crawford of Corfu, a very skilful practitioner was called, and to whom the attending physician relinquished the charge of the case and departed. Dr. Crawford diagnosed strangulated umbilical hernia, and prescribed opium, hot fomentations, etc. The use of the taxis proving unsuccessful, he advised further counsel, suggesting the probabilities of an operation; and, in obedience to a telegraphic summons, I visited the patient at 10,30 o'clock, P. M.

Finding a tumor of the umbilicus, about the size, shape, and "feel" of a large turnip, which was still the source of great pain, we administered half a grain of Sulph. Morphia, supplimenting it with inhalations of chloroform, hoping thus to control or abridge the sufferings of our patient in order to obtain a further examination of the tumor. After some delay this was accomplished, though with no little difficulty, on account of the *embonpoint* of the patient and the great tenderness of the swelling, when Dr. Crawford's diagnosis was confirmed. The remote symptoms were not yet urgent, so we decided to postpone the further consideration of operative measures, an alternative which now seemed inevitable, until morning, in order to obtain the advantage of daylight; meanwhile continuing the hot applications to the entire abdomen. Morphia was also given in half grain doses, at intervals varying from half an hour to two hours, throughout the night, supplimented, as in the first instance, with inhalations of chloroform at frequent intervals, to allay the very anguish of misery in which the patient was tossing about the bed whenever its use was suspended for a lengthened period. Nausea, which had existed from the first, towards morning became a troublesome factor, and finally enormous quantities of nearly pure bile were ejected, and the patient complained of feeling sick "all over." Morning, at last, brought an ominous relief from pain and great prostration withal, but the condition of the hernial tumor was unchanged, it still feeling as dense, hard, and inelastic as ever.

Deeming further delay as now inexcusable, at 9 o'clock, A. M., twenty-six hours after the seizure, the patient was etherized, and Dr. Crawford assisting me, I operated by gastrotomy in the *linea alba*, at the lower margin of the tumor. I first endeavored to relieve the stricture outside the sac, but failing in this the sac itself was opened, by a small incision, when an ounce or more of serum escaped; its contents were found to consist of omentum, intestine, and a portion of the liver had also thrust itself into this insatiable maw. Passing the finger into the opening and down to the seat of the stricture, which had a sharp, cutting "feel," a guide was thus made for the passage of an ordinary hernia knife, with which the structure was readily relieved by division obliquely downwards to the left of the *linea alba*. The external wound, not larger than three inches, was closed by two deep interrupted sutures, and dresssed in the usual manner. Bile was largely vomited during the first hour following the operation, after which it ceased altogether; union by adhesion resulted, and in two weeks, she was upon her feet and in the enjoyment of her usual health. It is proper to add that menstruation ensued three days subsequent to the operation.

Remarks:—Two or three points of interest in this case seem to demand notice before dismissing it altogether.

The pain, it will be observed was of an unusually severe character from the very outset, resisting every effort to relieve it unaided by chloroform, and requiring the almost constant exhibition of that drug, after the first twelve hours. The frequent vomiting of large quantities of pure bile is also a noticeable circumstance, accompanied as it was by the general *malaise*, which the patient often alluded to, as causing her to "feel sick all over." She explained her use of the expression, saying, that heretofore whenever the hernia had been incarcerated she had not felt as now—that in addition to the pain about the swelling, she experienced an indescribable misery, an unusual pain reaching through to the spine—that she felt sick "all over" as she had never felt before. The incarceration of a portion of the liver within the hernial sac becomes a feature of peculiar interest in view of the infrequency of its occurrence; and this no doubt offers an explanation of the strange and unusual feelings of the patient, and accounts as well, for the large quantities of bile ejected. The rapid recovery, without untoward symptoms, seems due, in a large measure, to the fact that the operation was made early, before the vitality of the parts became seriously impaired, and particularly before the patients strength became exhausted. My only regret is that we did not see, and operate upon this patient in the P. M. of the day before, thus abridging her sufferings by several hours.

Sir James Paget in a lecture upon hernia very aptly remarks, "that for every case in which an operation has been avoided by waiting, there have been two cases in which lives have been lost by waiting too long." Mr. J. Cooper Forster in "The Lancet" for February 1872, says, "two cases (of umbilical hernia) came under my care not so very long ago in which the medical gentleman in attendance showed an amount of intelligence which was quite delightful. I saw both of the cases within four hours—one within two—of the descent of the hernia, and in both I operated within an hour of seeing them, and they both got well—a very unusual thing in cases of umbilical rupture." Again, Mr. Le Gros Clark remarks (in the British Medical Journal, April 1872,) "if gentleness and forbearance were more generally adopted in applying the measures undertaken to afford relief, and purgatives and procrastination—often self-inflicted—were eliminated from the treatment, I believe that the statistics of the operation for strangulated hernia would present a far more favorable aspect than is at present the case."

I am well aware that it is not always possible to draw a sharp line between the laying down of other remedial measures, and the taking up of the knife, but a considerable observation has convinced me, as I presume it has most of you, that failures in the operation for strangulated hernia are mainly due to procrastination. While I would not condemn the judicious and moderate use of the taxis in appropriate cases, I am yet of the opinion, that as much harm as good comes of it, in the long run. It is a method, at once, very potent for good, and very powerful for harm, its employment summoning all the attributes of the clever surgeon—patience, courage, caution, firmness, gentleness, skill—these and perhaps others which I have not mentioned will be necessary, and then, unhappily, its use will be looked back upon regretfully in many instances.

Fortunately in the case related, the taxis had been employed moderately and with skill by my colleague, leaving nothing for me to determine in regard to it, after my arrival.

LITERATURE.

Most writers on umbilical hernia appear to pass over the subject very carelessly, and whoever attempt an examination of the literature of this form of rupture, will be confronted at the outset by the fact that much less attention has been paid to perfecting our methods and procedures in respect to it, than to the other varieties of the disease. This is due, no doubt, largely to the fact that it is of much less frequent occurrence than the inguinal or femoral forms of rupture; though Sir Astley Cooper says that if he had followed the orders of frequency of occurrence in his description of the several species of hernia, he should not have hesitated to place the umbilical next in order to the inguinal.

The earlier authors of treatises upon Surgery, speaking generally, devote more space to the consideration of this subject, than do those writing at a later day and within our own time. Mr. Samuel Cooper in the second decade of the present century, occupies eighteen pages in the discussion of *exomphalos*, while Mr. Liston and Mr. Dewitt, writing one and two decades later respectively, dismiss the subject with a few short paragraphs. Mr. Fergusson writing about the same time as Mr. Dewitt, makes no mention specifically of umbilical hernia. Sir Astley Cooper* writing in the beginning of this century, discusses the subject in his masterly style in a chapter of forty pages. But of all the writers of the first quarter of this eventful century for medicine, Sir William Lawrence† has given us, in a chapter of twenty-six pages, perhaps, all things considered, the most clear, concise, and scholarly account of the malady under consideration. I have, in the course of this paper, quoted his opinions and sayings largely, as indeed, I have most of the other authors mentioned.

In our own country the venerable and ever to be venerated Gross, in his admirable treatise on Surgery, has devoted as much space as was consistent with the comprehensive nature of his work, to a succinct and practical detail of the more salient features of umbilical hernia; while Hamilton writing about the same period, gives his views in a few trite and terse paragraphs, extending through a page and a half of his unique and valuable volume—epigrammatic in style and full of practical teachings.

These, and other authors—contemporaneous with them, but whom I must omit to mention specifically for lack of time, may be examined with pleasure and profit in the investigation of this interesting subject.

CAUSES.

The causes of umbilical hernia in the adult, may be, in the main, classed under three heads: First, pregnancy and laborious parturition; Second, obesity; and Third, straining at stool.

It is more often a disease of woman; and pregnancy, with its attendant phenomena, is the most common cause of the malady in the adult female.‡ This cause operates chiefly in the more advanced months of gestation, the mechanical pressure of the gravid uterus, as it rises upwards, carrying the intestines before it, encroaches upon and narrows the space appointed for the abdominal viscera, thus aggravating the complaint if it has already existed, or

* See Sir A. Cooper on Hernia, page 259, et seq.

† See Lawrence on Ruptures. Am. Ed. 1843, p. 406, et seq.

‡ Sir A. Cooper on Hernia. Ed. 1844. p. 263.

becoming the source of it, as the case may be ; and the tumor generally becomes larger with every pregnancy, as might readily be anticipated. "Women often also ascribe this disease to laborious parturition."*

Sir William Lawrence† remarks that "the distension of the *linea alba* by the gravid uterus so strongly favors the occurrence of *exomphalos*, that the number of females afflicted with this rupture greatly exceeds that of males."

The second most common cause of this malady in the adult is obesity. According to Mr. S. Cooper,‡ dropsical and corpulent individuals of both sexes are frequently seen afflicted, and Sir William Lawrence§ alludes to the fact that excessive corpulency in both sexes acts, by weakening the navel or the immediately surrounding fibres of the *linea alba*, to produce umbilical hernia. And again, Sir Astley Cooper‖ declares that a "very frequent cause of this complaint is an extraordinary degree of obesity, which, by enlarging the omentum and mesentery, renders the abdomen less capable of retaining its contents."

Straining at stool is referred to by Gross as one the causes which operate to produce umbilical hernia in the adult, though, I opine, it is a very infrequent one. It is, however, highly probable that any sort of straining, excessive in degree, as in pulling or pushing at a heavy load, or lifting a weighty burden, may, in a previously weakened or imperfect state of the parts, operate to protrude the abdominal viscera at or near the umbilicus.

### STATISTICS.

The statistics of hernia, always full of interest to the profession in general, form a favorite field of study for the practical surgeon, and it may not be out of place to introduce here, such as bear more especially upon the branch of the subject under consideration.

During a period of 28 years, the City of London Truss Society relieved 83,584 cases of rupture, of which 3,439, or a fraction less

*Sir A. Cooper on Hernia. Ed. 1844, p. 263.
†Lawrence on Ruptures, 1843, p. 411.
‡First Lines in Surgery, 1822. Vol. II, p. 14.
§Lawrence on Ruptures, 1843, p. 410.
‖On Hernia, p. 263.

than 1 in 25, were of the umbilical variety. Of these 3,439 cases of umbilical hernia, 664 were men, and 2,275 were women—more than four times as many wcmen as men. Of 2000 ruptures observed by Monikoff, 78 were exomphali.* Camper saw at Amsterdam, 1,968 cases, of which only ten were umbilical hernia. Of seventy-one cases reported by Soemmering, in Holland, (all umbilical hernia,) seventeen were men and fifty-four were women.

In Vol. III, St. Thomas Hospital Reports, 1873, Mr. Croft gives the statistice of 2,401 cases of hernia, of which 104, or one in twenty-three, were umbilical ruptures, and of this number forty-two were males, and sixty-two females.

The Medical Statistics of the Provost Marshal General's Bureau, compiled by Surg. J. H. Baxter,† furnish some interesting data in this connection. An examination of table XIX of this report exhibits the fact that of 334,321 "recruits, substitutes, drafted, and enrolled men of various nativities," 17,296 were rejected on account of hernia, a ratio per 1000 of 50.554. Of the 17,296 rejected for hernia of all kinds, 317 were so rejected by reason of umbilical rupture, a ratio per 1000 of .948. For the purpose of showing the relation umbilical hernia bears to the white and colored races respectively, I have extracted from table XVII (p. 433, vol. II,) the following data. Of 341,569 Whites, Colored and Indians, examined 306 were rejected on account of umbilical hernia, 315.620 were White, of which number 125 were rejected for umbilical rupture, a ratio per 1000 of .396.

25.828 were Colored, and 181 rejected, a ratio of 7.008 per 1000.

121 were Indians, and none were rejected for this disease.

The fact that umbilical hernia is more frequent in the Colored race than in the White, as here shown, is probably owing, in part, to want of care in adjusting the umbilical bandage after birth,‡ and also, in part, to the fact that the negro babies are more neglected during infancy; they being allowed to cry excessively before the abdominal parietes become perfected.§

---

*Sir W. Lawrence, p. 24.

†Washington Gov't Printing Office. 1875.

‡Med. Statistics, P. M. G. Bureau, Vol. 1. p. 349.

§Med. Statistics, P. M. G. Bureau, Vol. 1, p. 372.

ANATOMY.

A few of the anatomical characteristics of umbilical hernia may with propriety be noticed now. The shape and size of the tumor are variable, according as the subject is obese or the reverse; in the fat subject it is spherical, broader at its base and less prominent; in the thin person it is usually free and pendulous. These hernia sometimes attain an enormous size, particularly may they do so in women, who, from having borne many children, have pendulous abdomens.

It was formerly supposed that *exomphalos* was devoid of a true hernial sac, many of the older surgeons of great renown having denied its existence. But it has been clearly demonstrated that umbilical hernia is not only furnished with a true peritoneal sac, but it possesses also a more superficial investment, derived from a condensation of the surrounding cellular tissue.* It is, however, often the case that the coverings of this hernia are so thin, and particularly in old cases, portions of the sac become so absorbed as to make it difficult to trace on the front of the tumor; circumstances which should caution the surgeon in operating for umbilical hernia; otherwise he may wound the intestine with the first stroke of the knife. In *exomphalos* the sac usually contains both omentum and intestine, rarely intestine alone, and still more rarely the liver or other abdominal viscera.

Sir Astley Cooper† asserts that he has never seen the umbilical hernia in the adult, but that it contained omentum, though Sir William Lawrence reports a case upon which he operated with success, which contained small intestines only.‡

"The transverse arch of the colon is the bowel most frequently protruded; but the small intestines are also frequently found in the sac, and in some unusual instances the coecum."§ Mr. Holmes states that he has seen nearly the whole intestinal canal in the sac enveloped by omentum.

DIAGNOSIS.

The diagnosis of umbilical hernia cannot, ordinarily speaking, be attended with great difficulty. It usually begins as a very small

*Lawrence on Ruptures. p. 409.
†Sir A. Cooper on Hernia, 1844, p. 261.
‡Lawrence on Ruptures, 1843, p. 411.
§S. Cooper, First Lines, 1822. Vol. II, p. 14.

tumor at the navel, which is easily returned, but re-appears upon removing the pressure and directing the patient to cough. As it increases in size it gravitates and becomes pendulous in the thin subject, and its boundaries can be readily defined. In the obese individual, however, the case is not quite as simple, since the tumor may become buried in the fat, and extend itself between the integument and muscles, without causing external swelling. Gross relates a case of this sort which became strangulated, and was overlooked, but the autopsy proved it to have been the cause of death.

Sometimes, says Mr. S. Cooper,* a tumor grows from the navel, and puts on the appearance of an umbilical hernia ; also a large collection of hydatids within the liver, or upon its surface, sometimes give rise to a tumor at the side of the navel, which is liable to be mistaken for an *exomphalos*.

" Scarpa," says Mr. S. Cooper, " lays down with great accuracy, the distinguishing characteristics of both the true umbilical hernia, and of other cases which occur in the *linea alba* near the navel. The first disease, says he, whether met with in the infant or the adult, has a circular neck, or pedicle, at the circumference of which the tendinous margin of the umbilical ring can be felt with the end of the finger. Whatever may be the size of the tumor, its body always retains nearly a spherical shape ; nor can any wrinkle of the skin, nor anything at all resembling the cicatrix of the navel be observed, either upon the convexity or upon the sides of the swelling, the skin being a little paler and thinner at some points than others; on the contrary, in a hernia of the *linea alba,* the neck of the swelling is of an oval shape, like the fissure through which the protrusion has taken place. The tumor itself is also constantly of an oval form. When the finger is pressed deeply round its neck, the edges of the aperture in the *linea alba* are perceptible ; and if the hernia be *very near* the navel, the umbilical cicatrix may be seen on one of the sides of the swelling, a *sure indication* that the viscera do not protrude through the umbilicus itself."†

*First Lines. 1822, p. 12.

†Cooper's First Lines. Ed. 1822, Vol. II, p. 11.

Before the time of Petit, as I have before remarked, all umbilical hernia were supposed to protrude through the opening of the navel. While asserting this fact still to be true with regard to infants, he says: "I would not wish to be understood to assert, that, in adults, the parts never issue out of the umbilicus; but as I have seen this sort of case only twice in my life, this small number compared with the great opportunities I have had for seeing umbilical hernia, authorizes me to state that out of one hundred of these ruptures, not two happen through the opening of the navel, but the protrusions take place above, below or on one side of that part."* Mr. S. Cooper believes that in adults, the protrusion happens more frequently above than below the umbilicus, which, according to Scarpa, is due to the fact that the upper half of the *linea alba,* extending from the xyphoid cartilege to the navel, is naturally broader and weaker than the lower half, while the recti muscles also become situated nearer together as they descend from the navel to the pubes.†

PROGNOSIS.

When the umbilical hernia becomes strangulated it is the almost universal opinion among authors, that the prognosis is much more unfavorable, than in the inguinal or crural varieties. "Several of the best practical writers all concur," says Mr. S. Cooper, "in one important statement, which a prudent surgeon should constantly recollect, viz: that the *exomphalos* and hernia of the *linea alba* are less subject to true strangulation than the generality of other ruptures; but, that when it unfortunately takes place, the symptoms are more intense, and the accession of gangrene more rapid than any other species of hernia."‡

M. Demarquay, says, in a paper published in the *Bulletin de Therapeutique* for Oct. 30, 1874, that he has never seen recovery follow the usual operation performed after attempts at reduction, which are ordinarily futile in consequence of the numerous old adhesions.

---

*Petit. Treatise on Surgical Maladies. Vol. II, p. 250.
†First Lines Surgery. Ed. 1822. Vol. II, p. 11.
‡First Lines. Ed. 1822. Vol. II, p. 16.

This unusual fatality may be accounted for, in part, because of the subjects more commonly affected, such persons being unfavorable for operations; and in part, because of the general intestinal disorder, which exists with, or is speedily produced by the rupture; and again, in part, because of the proximity of the stomach, to which irritation and inflammation are readily propagated; but more particularly, as I opine, because of the too frequently great and inexplicable delay in making the operation for the relief of these cases.

### TREATMENT.

The treatment of umbilical hernia in the adult resolves itself under three different heads, corresponding to the three several conditions under which the malady may be observed; that is to say, whether it be in the reducible, irreducible, or strangulated state. To the relief of the latter condition we shall deem it proper to restrict our observations at this time.

Now, as always, the chiefest factors in the treatment of strangulated hernia, are the taxis, and the knife. Whatever other therapeutic measures may be resorted to, can only be regarded in the light of aids to these two potent remedies; nor is it too much to assume, that their timely and skilful employment will bring successful results, in the vast majority of cases. The first object, then, in the treatment of a strangulated umbilical hernia, as indeed is the case in other varieties, is to attempt its reduction by the taxis. So much has been written and said of late in regard to the methods of its employment, that it would be a mere work of supererogation on my part, to enter into an extended detail of when and how to use the taxis; neither would it comport with the scope and limits of this paper to do so. But I may with propriety suggest a single rule of action which will apply to the vast majority of strangulated *exomphali,* viz: to make, under chloroform, one decided effort with the taxis, and be prepared to operate if it should fail to reduce the hernia; for nothing does more harm in such cases than repeated unsuccessful attempts at reduction. It has become a rule now-a-days with many eminent surgeons, never to employ the taxis except with an anæsthetic; for they believe that more harm than good comes of its use, unless all muscular resistance by the patient be put out of the question. It would be well, in my humble opinion, if this principle was more universally taught in our schools. I need not stop to condemn prolonged attempts at reduction—the violent or forcible use of the taxis in the "reduction at all hazards" plan—since it can have no place in the treatment of strangulated umbilical hernia; for surely here, if anywhere, should the surgeon be gentle and self-restraining in handling the delicate structures, ever mindful of the fact that he may do them infinite harm.

In case the tumor be inflamed, with tender and painful coverings, the taxis will be wholly inadmissible, and the case passes at once under the domain of the knife. If perchance the symptoms either remote or local, be not urgent and some delay becomes advisable, while waiting it may be proper to make use of hot fomentations, enemata, or opiates; but the tobacco glyster of Sir Astley Cooper; gymnastic postulations with the head downwards and the feet "in the air;" cupping glasses and puncture of the intestine; these and other like and unlike things which have been so aptly styled by Sir James Paget as "ingenious wrong doings, more dangerous than the operation which they are intended to avert," may be put aside as worse than useless in the management of the condition now under consideration.

"Purgatives" adopting the language of Sir James Paget, "I believe, had better not be thought of, if there be any marked signs of strangulation. There are no clear indications for determining the cases in which they might possibly be useful; and if they do no good, they may do grievous harm."

The operation for strangulated umbilical hernia has been attended with great and unusual fatality from the earliest period of its history. Sir W. Lawrence affirms * that "the greatest practical writers have strongly represented the frequent fatality of the operation for strangulated exomphalos, and declares that the results of his own experience coincide entirely with their statements." He further asserts that the majority of operations he has made himself, or seen done by others, have ended fatally. There appears to have been, among the older surgeons, a great dread of the early operation, for they pretty generally advise delay, and constantly

* Lawrence on Ruptures, Ed. 1843, p. 428.

warn of the probably fatal termination of cases in which operative measures are finally resorted to. Scarpa however strongly opposes this doctrine and advised that the operation be made early; attributing its ill success to its being performed too late. Sir Astley Cooper seems to have concurred in the opinions of Scarpa, judging from his practice, but he does not speak emphatically in regard to the matter. Authorities in our own time do not differ from those referred to in regard to the fatality of the operation. Hamilton declares * that the statistics of this operation present a more unfavorable result than do the statistics for either inguinal or femoral hernia. He further asserts that when it has been necessary to open the sac the result has generally been fatal. Gross says † "when we consider how disastrous have been most of the operations that have hitherto been performed for the relief of strangulated umbilical hernia, we can scarcely lay too much stress upon the protracted and judicious employment of the taxis." That there are certain circumstances peculiar to this variety of rupture which render the operation often unsuccessful, cannot be doubted, the large size of the tumor, its free and direct communication with the abdomen, the immediate proximity of the stomach, and the condition of the patients who often suffer with the malady, are all unfavorable to success, and surround the operation with peculiar dangers. These, however, seem to me, arguments in favor of the early operation, rather than reasons for procrastination. To jeopardize to results of an operation, already singularly hazardous, even though with but a single jot of additional danger ought to be reprehended; and I am sure that in these days of improved abdominal surgery the statistics of this operation can be brought up to a much better showing than unhappily is at present the case, if the knife be appealed to earlier than has been the usual practice amongst surgeons, both ancient and modern.

Says Colles, "there is something peculiar in these hernias that causes peritoneal inflammation sooner than others; they are extremely different from all other hernias, and in nothing more so than in the severity of the mischief arising from them, and the

---

* Prin. and Practice of Surgery 1st Ed. p. 744.
† System of Surgery, p. 714.

rapidity with which they run their course." Sir Astley Cooper mentions a case which run its course and ended fatally 17½ hours from the commencement of strangulation.

As to the method of the operation little need be said, since one may cut up, or down, or sidewise and not be wrong; for the danger in this operation, according to Sir Astley Cooper, is in wounding the intestine, and not the blood vessels.

In the case reported, I chose the lower margin of the tumor for the incision as being more convenient and simpler in execution. In a larger and more pendulous hernia, the upper side would possibly be preferable, though I should say, speaking generally, that an incision through the abdominal walls in such immediate juxtaposition to the stomach is to be avoided if possible. Since the mouth of the sac is always, according to Sir James Paget, the seat of the stricture, the middle of the first incision may, with propriety, be right over it; and it may be made at the most convenient point upon the circumference of the tumor—above, below, or to one side, as the case may be—an opening from two to three inches long being usually sufficient. Caution should be observed in making the first incision, lest the intestine, owing to the thinness of the external coverings, be wounded by the first stroke of the knife. When practicable, the stricture may be divided outside the sac, but this is generally difficult, for the sac is thin, and there may be little tissue between it and the fibrous ring.

Gross strongly advises division of the stricture without opening the sac, "experience," he says, "having shown that its division is fraught with the greatest danger, from its liability to be followed with fatal peritonitis"; but if the operation be made early, this liability will be greatly diminished. Sir James Paget suggests the propriety, in large umbilical hernia, of making two incisions at opposite borders of the ring. If the attempted division of the stricture external to the sac be unsuccessful—and the trial should not be prolonged, since it is a good thing to succeed in, but a bad thing to fail in—the sac itself should be opened by an incision large enough to admit the passage of the finger, along which an ordinary hernia knife is introduced, and the stricture divided according to the usual rules governing the operation for strangulated hernia. "In dividing the orifice of the hernial sac and the linea alba," says Sir Astley Cooper,* "there is no risk of cutting through any vessel of sufficient consequence to endanger life; for it is refining upon anatomy to mention the umbilical vein or arteries in this operation, as the latter two are mere cords, and the former, even if it were opened and divided, would be readily closed by a dossil of lint."

Strangulated umbilical hernia is liable to occur, as might be expected, during gestation; and pregnancy, according to Sir Astley Cooper, does not appear to add to the risk of the operation. Sir William Lawrence has operated successfully in the well advanced pregnant state, considering it not an objection, but rather an additional reason for rescuing the patient as quickly as possible from the imminent danger of her rupture.†

M. Demarquay, who has paid considerable attention to umbilical hernia of late, alludes, in the paper hereinbefore noticed, to the almost universally fatal issue of operations for strangulated *exomphali*, and describes a new operation by which he has been enabled to save one out of the four patients upon whom he has put into force. He extends an oblique incision from the middle portion of the tumor towards and on to the left side of the abdominal wall. He avoids the right side on account of the proximity of the umbilical vein; the middle line, also, as presenting an objection in the *linea alba.* The cellulo-adipose tissue is then divided, layer by layer, until the pedicle of the hernia sac is reached, when a small incision is made at the lower part of its left side; through this the left index finger is introduced until its end rests upon the orifice of the hernia. Along the finger a falciform bistouy is guided, and an incision is made implicating the left side of the circumference of the sac, and extending entirely through all the substance of the abdominal wall at this point. The aim of the operation is to relieve the strangulation by a *débridement* of three or four centimetres, and then if the intestine has not been too much compressed, and it, as well as the omentum, are still only in a state of congestion, the parts concerned will become disgorged and their functions

*Sir A. Cooper on Hernia. Ed. 1844, p. 294.
†Lawrence on Ruptures. Ed. 1843, p. 431.

re-established, even though there be a little herniary peritonitis. It is difficult, however, to understand what the precise advantage of this plan is, and particularly is this the case when we consider the large percentage of failures.*

Mr. Thos. Annandale proposes † a somewhat novel method of operating for the relief of strangulated hernia, particularly applicable, as he thinks, to *exomphalos* in the adult, especially if the coverings are thin or ulcerated, or the hernia large. To quote his words, "The principle of this method consists in making a small incision through the abdominal walls and opening the abdominal cavity *near* the hernia, instead of cutting into the hernial sac itself or exposing it. One or more fingers are then to be inserted into the abdominal cavity, and the protruded structures contained in the hernial sac drawn back from within." He applied this method to a case of umbilical hernia in a male aged 61 years, in which symptoms of strangulation had existed three days. He made an incision three inches long midway between the xyphoid cartilage and the navel, and opened the abdominal cavity to the extent of an inch and a half. The hernial protrusion consisted of several inches of small intestines adherent to the sac, but these easily gave way, and the whole contents of the sac were brought out through the abdominal wound and examined. He also freely divided the firm neck of the sac with a probe-pointed knife introduced through the wound, returned the intestines and closed the incision in the usual manner. Three hours after the operation the patients bowels opened freely; the next day he was weak, though free from pain; but, says the operator, the exhaustion continued, and he died somewhat unexpectedly (?) forty-eight hours after the operation.

But, this paper has already exceeded the proper limits, and I must bring it to a close, leaving many points of interest in this important subject still untouched.

Therefore, in conclusion, permit me to remark that, while no originality has been essayed in the presentation of this subject, I have, on the other hand, merely sought to draw attention to a somewhat neglected species of hernia, by massing some of the sa-

---

*See Med. Times & Gazette, Dec. 5th, 1874; and London Med, Record, March 17th, 1875.
†See Edinburgh Med. Journal, Sept. 1873.

lient features in the teachings, writings, and opinions of recognized authorities thereupon, and finally, to advocate, in general, the early operation for the relief of strangulated umbilical hernia in the adult.

